# Evaluation of the EMBOPIPE flow diverter device: in vivo and in vitro experiments

**DOI:** 10.1186/s41016-024-00360-9

**Published:** 2024-03-12

**Authors:** Yongnan Zhu, Fanyan Zeng, Jian Liu, Shiqing Mu, Ying Zhang, Xinjian Yang

**Affiliations:** 1 Department of Beijing Neurosurgical Institute, Fengtai District, Capital Medical University, No. 119, South Fourth Ring West Road, Beijing, 100070 People’s Republic of China; 2 Fengxian District, Heartcare Medical Technology Co., Ltd, Building 38, No. 356 Zhengbo Road, Shanghai, 200000 People’s Republic of China; 3 Neurosurgical Institute & Department of Neurosurgery, Fengtai District, Beijing Tiantan Hospital, Capital Medical University, No. 119, South Fourth Ring West Road, Beijing, 100070 People’s Republic of China

**Keywords:** Coating, Flow diverter, Phosphorylcholine, Rabbit aneurysm model, Thrombogenicity

## Abstract

**Background:**

Although flow diverter device (FDD) has brought revolutionized advances in endovascular treatment of intracranial aneurysms, it also presents considerable drawbacks as well, as the innovation for novel device has never stopped. This preclinical research aims to evaluate the safety and efficacy of a newly developed FDD, the EMBOPIPE, through in vivo and in vitro experiments.

**Methods:**

Aneurysms were induced in 20 New Zealand white rabbits which were randomized to three follow-up groups according to the time elapsed after EMBOPIPE implantation (28, 90, and 180 days). Additional EMBOPIPEs were implanted in the abdominal aorta to cover the renal artery in nine rabbits. Angiography was performed immediately after device placement in all groups. Aneurysm occlusion, patency of renal arteries, and pathological outcomes were assessed. For the in vitro experiments, we measured the thrombogenic potential of EMBOPIPEs (*n* = 5) compared with bare stents (*n* = 5) using the Chandler loop model. Evaluation indicators were the platelet counts, macroscopic observations and scanning electron microscopy.

**Results:**

EMBOPIPEs were successfully deployed in 19 of 20 rabbit aneurysms (95.0%). The rates of complete or near-complete aneurysm occlusion were 73.3%, 83.3%, and 100% in the 28-, 90-, and 180-day groups, respectively. All renal arteries covered by EMBOPIPEs remained patent, and the mean difference in renal artery diameter before and after the device placement in the three groups was 0.07 mm, 0.10 mm, and 0.10 mm, respectively (*p* = 0.77). Renal pathology was normal in all cases. The pathological findings of the aneurysms were as follows: thickened and adequate neointimal coverage at the aneurysm neck, minimal inflammatory response, near-complete smooth muscle cell layer, and endothelialization along the device. In vitro experiments showed that the platelet counts were significantly higher in EMBOPIPE blood samples than in bare stent samples and that platelet adhesion to the device was lower in the EMBOPIPE stent struts compared with bare stent struts through macroscopic observations and scanning electron microscopy.

**Conclusions:**

The EMBOPIPE can achieve high rates of aneurysm occlusion while maintaining excellent branch artery patency. It exhibited wonderful pathological results. This novel device with phosphorylcholine surface modification could reduce platelet thrombus attached to the stent struts.

## Background

Clinical experience with flow diverter device (FDD) has evolved since their approval in the United States, Europe, and other countries. FDD have significantly revolutionized the treatment of intracranial aneurysms. Unlike traditional intracranial stents, FDD have better metal coverage, higher pore density, and lower porosity [1]. These features lead to aneurysm occlusion after FDD placement due to modification of intra­aneurysmal hemodynamics and neointimal endothelialization [2]. However, these characteristics also pose considerable drawbacks in particular the strict requirements for dual antiplatelet therapy and the risk of ischemic complications after FDD placement [3]. According to several large studies on flow diverters, the incidence of ischemic stroke varies from 4.7 to 10% [4–8]. Several other safety concerns have also limited their use: potential occlusion of side branches or perforating arteries leading to ischemic complications, stent thrombosis, and delayed aneurysm rupture [9]. Thus, demonstrating safety and efficacy for novel FDD that either decrease device thrombogenicity or expedite endothelial growth is a priority.

**Fig. 1:**
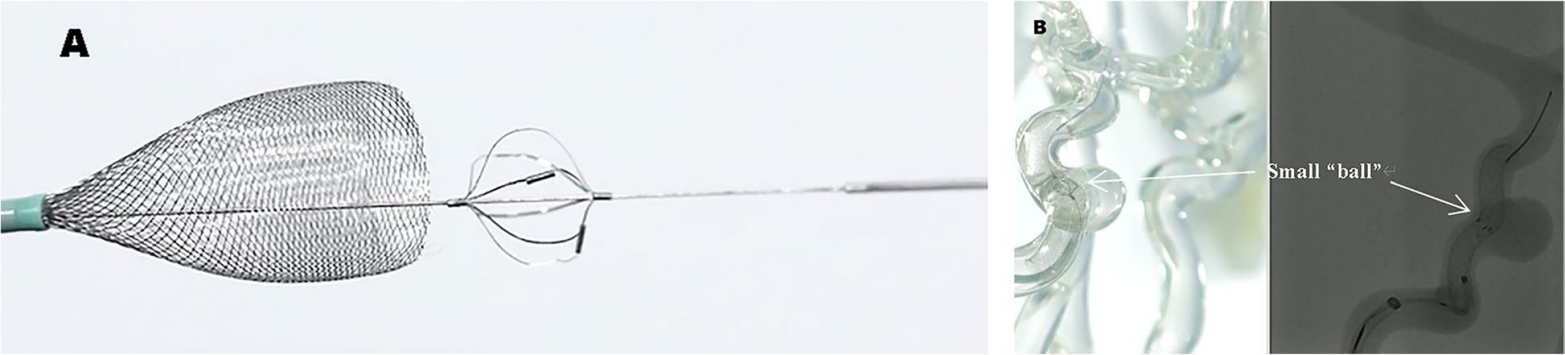
EMBOPIPE: model illustrations and digital subtraction angiography images. **A** The schematic of the “little ball,” which unfolds as the stent begins to deploy. **B** The “little ball” can massage the device after it is fully deployed. The four radiopaque platinum markers are clearly visible

The EMBOPIPE (HeartCare Medical Technology Co., Ltd, Shanghai, China) is a newly developed self-expanding stent constructed from 36 strands of cobalt–chromium alloy and 12 strands of platinum wire. The metal coverage is 32%, similar to the Pipeline Embolization Device (PED; Medtronic, Irvine, CA, USA; 30–35%) and Silk Vista (Balt, Montmorency, France; 35–55%). The novel delivery system includes a ball-like structure with a friction pad, which we call the “little ball.” It is located at the anterior end of the delivery guidewire and unfolds as the device begins to deploy (Fig. 1). It has four radiopaque platinum markers that could improve the visibility of the proximal stent. The “little ball” massages the scaffold after the device is fully released, without the need for guidewire exchange, and achieves better wall apposition. In addition, the surface of the cobalt–chromium and platinum strands that make up the device are covalently bonded to a synthetic phosphorylcholine polymer. This study aimed to evaluate the safety and efficacy of the EMBOPIPE in both in vitro and in vivo experiments.

## Methods

### In vivo* experiments*

The in vivo portion of the study was approved by the Institutional Review Board and conducted in the Animal Center (Xi Dian, Sichuan Province, China) in accordance with animal care experimentation guidelines. Elastase-induced aneurysms were created at the origin of the right common carotid artery in 20 New Zealand White rabbits (both male and female, weighing 3.0–3.5 kg), as previously described [10]. These rabbits were randomized to three follow-up groups: 28, 90, and 180 days after device implantation. The number of rabbits in each follow-up group was six, and another two animals were kept as spare cases. The required sample size was calculated using the unpaired *t*-test and based on the principles of economy and accuracy. The aneurysms were allowed to mature for at least 4 weeks prior to EMBOPIPE implantation. All rabbits received dual oral antiplatelet therapy with aspirin (10 mg/kg) and clopidogrel (10 mg/kg) from 3 days prior to device implantation until they were sacrificed. No rabbits were excluded, and no inclusion or exclusion criteria were determined.

After anesthesia, the right inguinal region of the rabbit was prepared and disinfected by an experienced surgeon. After isolation and exposure of the femoral artery, a 5-French vascular sheath (CERENOVUS, Johnson & Johnson, New Brunswick, USA) was inserted, and a 5-French angiographic catheter (CERENOVUS) was navigated to the aortic arch under fluoroscopic guidance. Nitroglycerin was administered to relieve vasospasm during the operation. Digital subtraction angiography (DSA) was used to confirm aneurysm creation and to measure the aneurysm neck, width (defined as the maximum dome diameter), and length (defined as the distance from the aneurysm dome to the neck). After establishing a roadmap angiogram, a microcatheter (HeartCare) was placed in the subclavian artery distal to the aneurysm using a microguidewire (Target Medical, Beijing, China). A preloaded EMBOPIPE was then introduced into the microcatheter and deployed across the aneurysm neck. Angiography was performed immediately to confirm proper placement and to assess aneurysm obliteration. Animals in all groups underwent repeat DSA under anesthesia by the same surgeon after device placement, for data collection, and were then euthanized. Aneurysms, parent arteries, renal tissues, and stented aortic segments were collected and immersed in 10% neutral buffered formalin.

Previous studies have confirmed that rabbit renal arteries are suitable for assessing branch artery patency [11, 12]. Therefore, to evaluate the patency of stent-covered branches, an additional EMBOPIPE stent was placed in the abdominal aorta to cover the renal artery fully in 9 nine rabbits. We evaluated renal organ damage and the mean difference in renal artery diameter before and after device placement to assess renal artery patency.

As previously described [13], the degree of aneurysm occlusion was graded as follows: grade I, complete occlusion; grade II, > 90% occlusion; and grade III, < 90% occlusion. Complete or near-complete occlusion was defined as grade I and II combined.

The pathological specimens were evaluated microscopically (Eclipse 50i; Nikon, Tokyo, Japan) and scored as follows: (a) extent of neointimal hyperplasia covering the aneurysm neck (1, thin neointimal hyperplasia [less than three layers of histocytes]; 2, thick neointimal hyperplasia [more than or equal to three layers]); (b) aggregation of inflammatory cells (0, no cells; 1, a few cells; 2, a mild number of cells; 3, a moderate number of cells; 4, a high number of cells); (c) percentage of intraluminal thrombosis (0, free of thrombus; 1, > 25% of vessel diameter; 2, 25–50% of vessel diameter; 3, 50–75% of vessel diameter; 4, > 75% of vessel diameter); (d) area of smooth muscle cell (SMC) apoptosis (0, no apoptosis detected; 1, punctate areas; 2, small areas; 3, medium areas; 4, large areas); (e) degree of vessel wall endothelialization (0, 0%; 1, < 25%; 2, 25–50%; 3, 50–75%; 4, > 75%).

### In vitro* experiments*

**Fig. 2:**
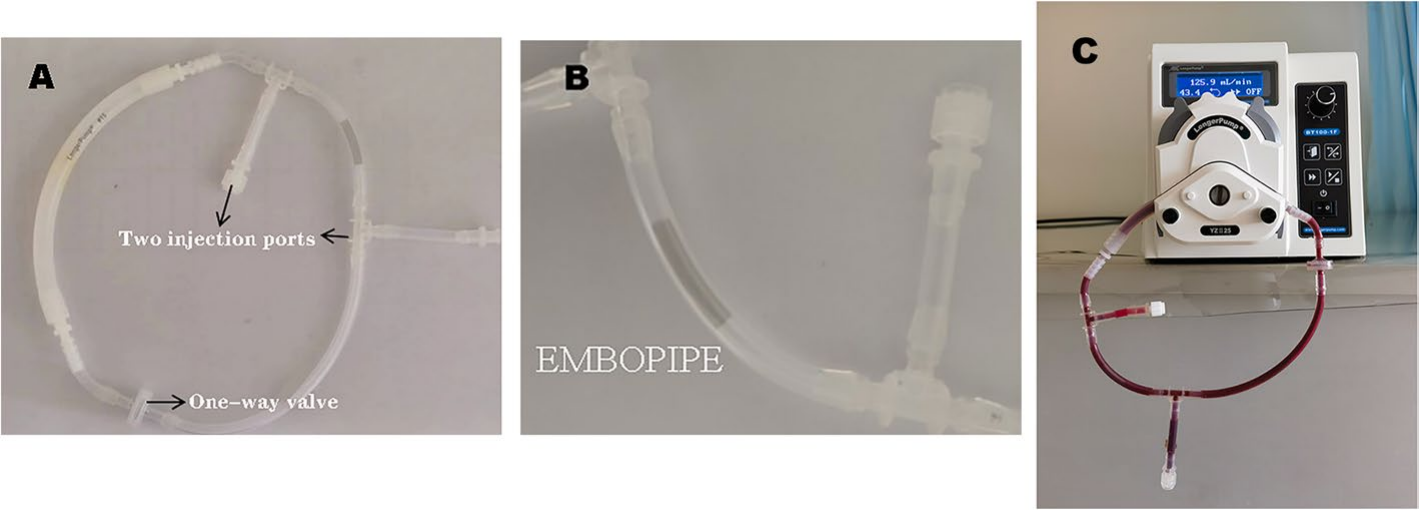
Experimental equipment used for the in vitro experiment. **A** Chandler loop tube containing an EMBOPIPE. Each loop consists of a one-way check valve and two injection ports. **B** A magnified view of the image in **A**. **C** The pump with a flow loop attached (containing a device and fresh human blood)

The in vitro experiments were approved by the Institutional Review Board. The antithrombogenic properties were compared between the EMBOPIPEs and the bare stents (HeartCare Medical Technology Co., Ltd, Shanghai, China) using the Chandler loop model, as previous reported (Fig. 2) [14]. The bare stent is a flow diverter device stent without a coating. The loops were fill with blood obtained from five healthy adult human volunteers. All volunteers provided written informed consent and had not taken any antiplatelet medication within the previous 2 weeks. We assess thrombogenicity quantitatively by using platelet counts and scanning electron microscopy (SEM).

We deployed five EMBOPIPEs and five bare stents into their respective Chandler tubes (medical grade polyvinylchloride; 4.61 mm internal diameter; approximate volume, 8.4 mL; HeartCare). Blood from each volunteer was used to test one EMBOPIPE and one bare stent. In addition, platelet counts were determined from each pre-experimental blood sample by using an automated analyzer. The blood-filled loop was connected to an extracorporeal circulation pump (BT100-1F Peristaltic Pump; Baoding Longer Precision Pump Co., Ltd, Hebei, China). The velocity was set at 100 mL/minute (to simulate human internal carotid artery flow) [15]. with a pump head setting of YZ2515. The total running time was 120 min. Then, the loop system was placed in a 37 °C water bath for the duration of the experiment. At the end of the experiment, the blood in the loop was collected and platelet counts again analyzed. The difference between the platelet counts before and after the Chandler loop experiment reflected the platelets that had aggregated and adhered to the EMBOPIPE/bare stent scaffold. The devices were also visually inspected and analyzed by SEM after fixation in glutaraldehyde.

### Statistical analysis

Statistical analyses were performed using the SPSS software version 25 (IBM Corp., Armonk, NY, USA). Pair-wise comparisons of aneurysm size and pathological parameters were conducted using analysis of variance (ANOVA). In vitro data were compared using the paired *t*-test. *P* < 0.05 was considered significant.

## Results

### Animal experiments

EMBOPIPE delivery and deployment was successful in 19 rabbits (95.0%). The only failure occurred because the parent vessel had a sharp bend (120°), and our experimental equipment was insufficient to pass the delivery system through his. Thus, the only remaining case was included in the 180-day group. Aneurysm neck, width, and length did not differ significantly between the three groups (Table 1).

**Table 1:** Aneurysm neck, width, and length according to group

Follow-up time	Neck (mm)	Wide (mm)	Length (mm)
28 days (*n* = 6)	1.98 ± 0.45	2.36 ± 0.20	4.68 ± 0.80
90 days (*n* = 6)	2.03 ± 0.33	2.71 ± 0.39	4.76 ± 0.50
180 days (*n* = 7)	2.36 ± 0.48	2.77 ± 0.28	4.89 ± 0.44
*P* value*	0.230	0.064	0.815

Data shown are means with standard deviation

^*^Analysis of variance

**Fig. 3:**
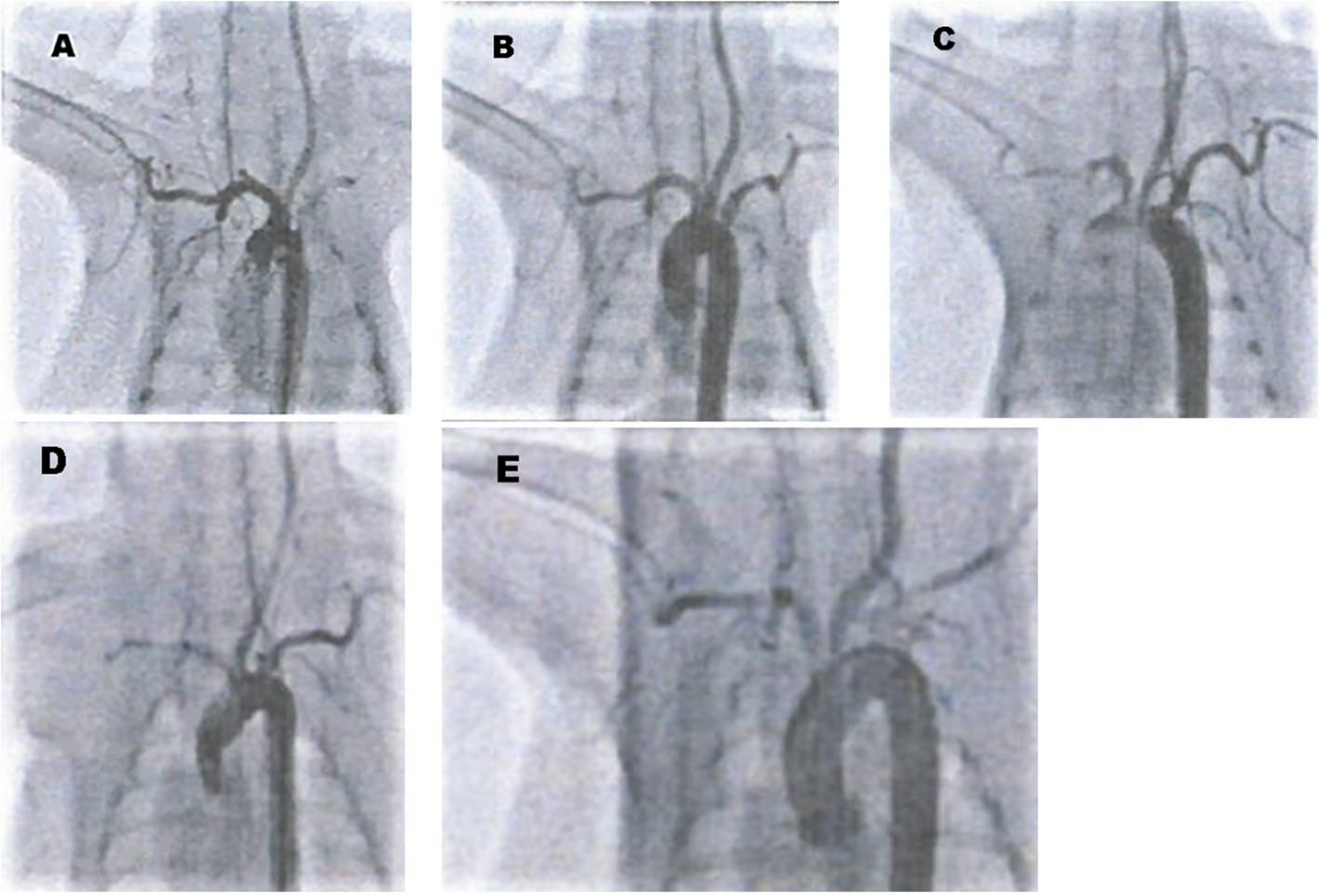
Representative digital subtraction angiography of the case before and after EMBOPIPE deployment in three groups. **A** The image shows successful aneurysm development. **B** Immediately image after the device deployment, the image shows proper stent deployment and prolonged intra-aneurysmal opacification. **C** Images taken 28 days after the device placement showed minimal residual aneurysm. **D**–**E** Two additional cases, at 90 and 180 days after stenting, respectively, showing that complete aneurysm occlusion had eventually been achieved

Immediate postprocedural angiography showed that all cases had prolonged intra-aneurysmal opacification after stent deployment. In the final angiographic follow-up images, all rabbits achieved complete or near-complete aneurysm occlusion (Fig. 3). Aneurysm occlusion was grade I in two (33.3%), grade II in three (50.0%), and grade III in one rabbit (16.7%) in the 28-day group; grade I in four (66.7%), grade II in one (16.7%), and grade III in one rabbit (16.7%) in the 90-day group; and grade I in six (85.8%) and grade II in one rabbit (14.3%) at in the 180-day group. The rates of complete or near-complete occlusion in the three groups were 73.3%, 83.3%, and 100%, respectively. None of the rabbits showed any neurological deficits during follow-up periods.

### Pathological results

**Fig. 4:**
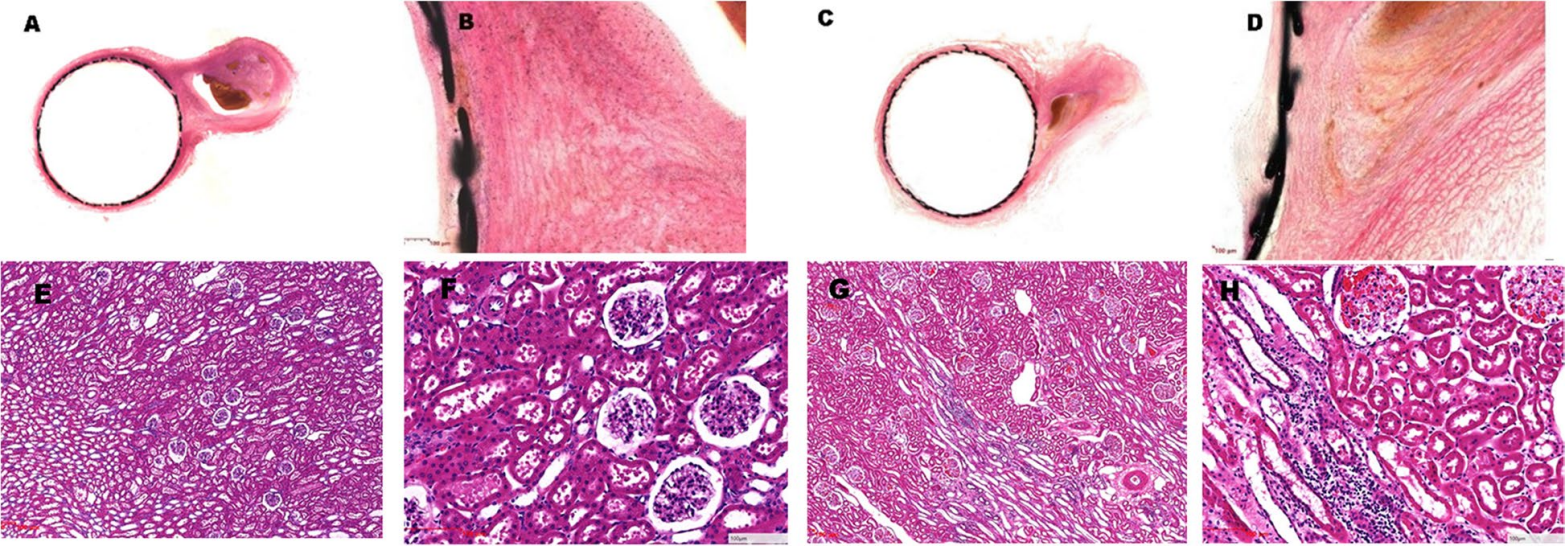
Representative pathological findings of aneurysm neck and renal organ tissue. **A**–**B** Thick neointima completely covers the aneurysm neck; endothelialization inside the device is mild and evenly distributed in the 28-day group. **B** shows a local magnification of the image in **A**. **C**–**D** The aneurysm was completely occluded 90 days after device placement. **E**–**H** Normal tissue organization found in the renal organ in 90-day (**E**–**F**) and 180-day (**G**–**H**) groups. **F**, **H** Higher magnification of **E** and **G**, respectively, shows that the morphology of the glomeruli and renal tubules is normal, and the cells are regularly arranged. **A**, **C** Hematoxylin and eosin (H&E)-staining images (0.62 ×). **B**, **D** H&E-staining images (5 ×). **E**, **G** H&E-staining images (2.5 ×). **F**, **H** H&E-staining images (10 ×)

Aneurysm neck specimens showed adequate neck coverage in all cases. Around the parent vessels, we found minimal inflammation, minor intraluminal thrombosis, and complete endothelialization along the stent struts. We observed a decreasing trend in detection of SMCs in the 90- and 180-day groups as compared with the 28-day group (*p* < 0.05; Table 2). The thick neointima completely covered the aneurysm neck at the 28-day follow-up, whereas the aneurysm was occluded entirely at 90 days after device placement (Fig. 4).

**Table 2:** Histopathological score results at 28, 90, and 180 days

Tissue section	Pathological pattern	28 days	90 days	180 days	*P* value
Aneurysm neck	Neointima cover	**1.83 ± 0.41**	**1.83 ± 0.41**	**2.00 ± 0.00**	**0.566**
Parent vessel	Inflammation	**1.17 ± 0.41**	**1.00 ± 0.00**	**1.00 ± 0.00**	**0.358**
	Intraluminal thrombosis	**0.00 ± 0.00**	**0.00 ± 0.00**	**0.00 ± 0.00**	**-**
	Deletion of smooth muscle cells	**0.67 ± 0.82**	**0.33 ± 0.52**	**0.00 ± 0.00**	0.032*
	Endothelialization	**3.67 ± 0.52**	**4.00 ± 0.00**	**4.00 ± 0.00**	**0.095**

Data shown are means with standard deviation

^*^Statistically significant (analysis of variance)

### Renal arteries and renal organ finding

All the renal arteries covered by the EMBOPIPE were patent on angiography. The mean difference in renal artery diameter before and after the device placement in the three groups was 0.07 mm, 0.10 mm, and 0.10 mm, respectively (*p* = 0.77). These mean values were not significantly different from each other, indicating that blood flow through collateral arteries covered by FDDs was not affected. In addition, all histopathological results of the renal tissue showed that the morphology of the glomeruli and renal tubules was as expected, with regular cell arrangement (Fig. 4), demonstrating that device placement had minimal impact on branch patency.

### In vitro* experiments*

**Fig. 5:**
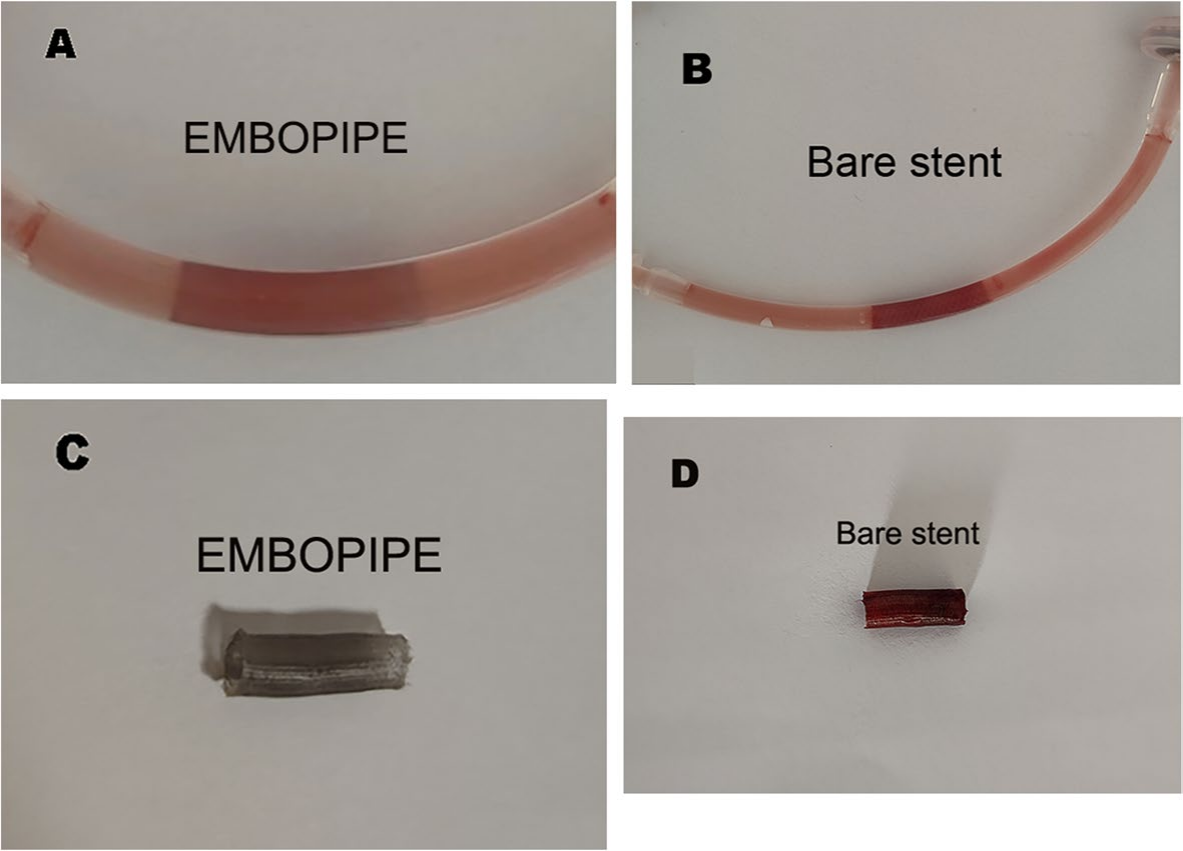
Representative images of thrombus accumulation on the scaffold. **A** The EMBOPIPE is shown in the Chandler loop. **B** The bare stent is shown in the loop. **C**–**D** Both devices are shown after removal from the loop. The bare stent shows more thrombus accumulation

Grossly, the surface area of the thrombus was less with EMBOPIPE than with bare stents (Fig. 5).

**Fig. 6:**
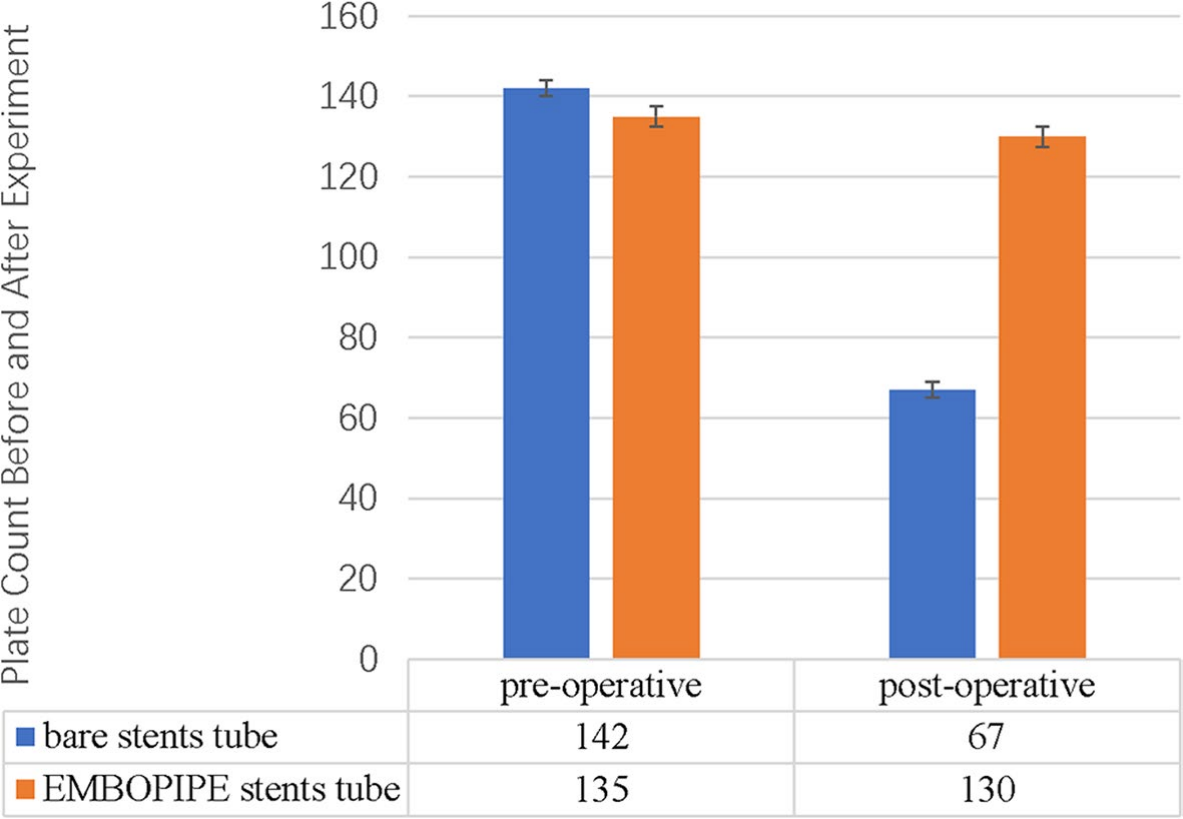
Mean platelet count (× 10^3^/μL) measured before and after the experiment

The mean platelet counts before and after the experiment are shown in Fig. 6. The mean difference was significantly higher for the bare stents ([75 ± 23)] × 10^3^/μL vs. ([5 ± 32)] × 10^3^/μL; *p* < 0.001).

**Fig. 7:**
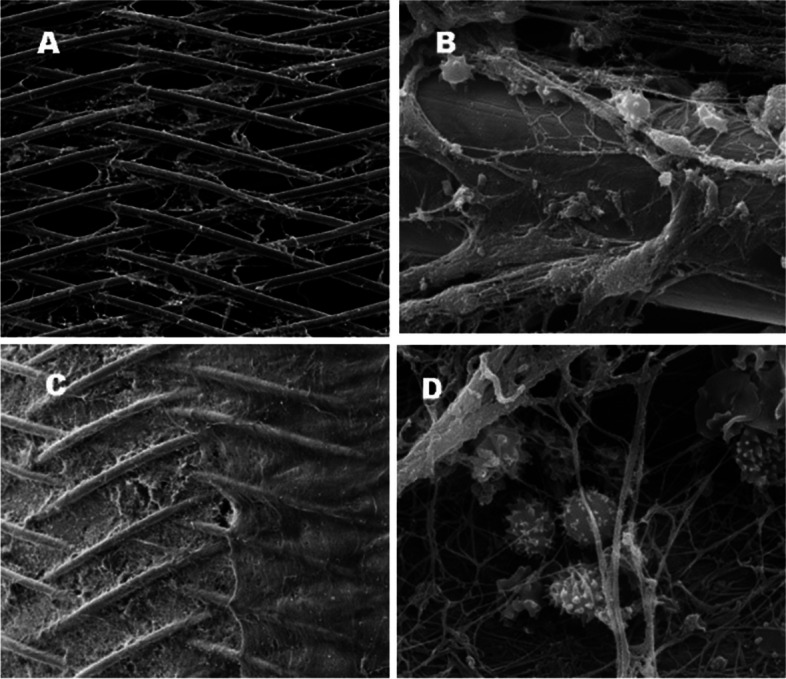
Representative high-resolution scanning electron microscope images of thrombus accumulation on the device surface. **A**–**B** The EMBOPIPE (100 × and 2000 × , respectively) showed no obvious platelet thrombus on the stent struts. **C**–**D** The bare stent (100 × and 2000 × , respectively) shows obvious thrombus accumulation covering the stent struts

SEM analysis of the thrombus showed apparent cellular and proteinaceous accumulation on the bare stent surface; in addition, the struts were almost completely obscured by the thrombus, which formed small agglomerates on and between the single wires. In contrast, no platelet thrombus was evident on the EMBOPIPE stent struts, and the wires were clearly visible (Fig. 7).

## Discussion

This study demonstrated the safety and efficacy of the EMBOPIPE in an elastase-induced rabbit aneurysm model and a Chandler flow loop model. The technical success rate of stent deployment was 95.0%, likely due to the novel self-expanding delivery system. The “little ball” and friction pad on the guidewire allowed easy release of the device and massage of the inner wall of the device after deployment to improve wall apposition without guidewire exchange. No other devices were required for EMBOPIPE deployment.

### Aneurysm occlusion

Aneurysm occlusion rate represent the effectiveness of treatment with FDD. The EMBOPIPE achieved complete or near-complete occlusion in 83.3% of the aneurysms in the 90-day group and complete occlusion in all animals (100%) in the 180-day group. This rate was comparable or even higher than that reported in previous animal aneurysm studies using other types of FDDs. In a 2016 systematic analysis, a total of 25 rabbit animal studies was included, and the 3-month rate of complete or near-complete occlusion among 68 rabbit elastase-induced aneurysms was 73.5% (95% confidence interval, 61.9–82.6%) [12]. In the same year, Kim et al. disclosed a novel FDD (FloWise, Angiovention, Seoul, South Korea) [11]. Similarly, they treated 31 rabbits with Elastase-induced aneurysms, and the rate of complete or near-complete occlusion was 100%.

### Branch arteries

FDDs may induce hemodynamic changes that contribute to reduced blood flow in FDD-covered vessels, due to their higher metal mesh density theoretically. The patency of side branches covered by FDDs is therefore controversial. A 2023 meta-analysis by Liu et al., which included 57 studies and 3789 patients with intracranial aneurysm treated with FDD, showed that the risk of branch occlusion-related complications was low (incidence rate < 5%) for the branches evaluated [16]. Similarly, studies in rabbits have reported no adverse events due to branch occlusion [12, 17]. Overall, the risk of such occlusion in existing studies appears to be low, which is consistent with the results of this study. The strengths of our study were the normal pathological results in target organs (renal) of the branch artery. In addition, we report data at the end of the study with up to 180 days of follow-up, which were less addressed by the previous studies.

### Histological findings

Endothelialization along the stent struts and neointimal coverage of the aneurysm neck promotes aneurysm occlusion [18]. Our histological findings showed a completely marked endothelialized neointimal coverage of the aneurysm neck within the EMBOPIPE early in the 28-day group and even thicker neointimal layer in the 90- and 180-day group. The excellent occlusion rates of EMBOPIPE be explained by this result. Matsuda et al. reported that surface-modified stents resulted in the development of more concentric neointima than did bare stents [19]. Their pathological findings were similar to ours. However, they did not evaluate the neointimal coverage at the aneurysm neck. A similar study of novel FDD (DiVeRt, Merlin MD Pte Ltd, Admirax, Singapore) also used a semi-quantitative scoring system to quantify tissue histopathology [20]. In their study, the score of neointimal hyperplasia covering the aneurysm neck was 3.98 ± 0.13 and also reached excellent aneurysm occlusion rates.

Kadirvel et al. investigated mechanisms of aneurysm healing after FDD treatment [21]; they concluded that endothelialization is dependent on an underlying smooth muscle cell substrate. Thus, the progressive loss of vascular SMCs and the aggregation of inflammatory cells play an important role, not only in the development of intracranial aneurysms but also in delayed aneurysm healing [22]. We rarely observed inflammatory cell aggregation, and most treated aneurysms showed an intact smooth muscle structure. In the few cases that did not, the loss of vascular SMCs showed a decreasing trend with increasing follow-up duration (*p* < 0.05), probably because they were able to secrete quantities of extracellular matrix and repair themselves.

### Thrombogenicity

Thromboembolic events following FDD placement are a clinical concern. Therefore, dual antiplatelet therapy is recommended, but this may also lead to bleeding complications [9]. Applying biomimetic phosphorylcholine surface modification has markedly improved stent hemocompatibility [23]. In our study, the platelet count results indicated that the EMBOPIPE induced less platelet aggregation than did the bare stent. Similarly, SEM showed that the EMBOPIPE was antithrombogenic. Keiko et al. investigated the thromboresistance of another surface-modified device, the FRED X (MicroVention Inc., Aliso Viejo, CA, USA), in vitro [24]. On the one hand, they found that the platelet count was lower with the FRED (a bare stent) than with the FRED X. On the other hand, SEM imaging showed that the accumulation of blood cell-like deposits was lower with the FRED X. In addition, another study found that a hydrophilic polymer coating significantly reduced the thrombogenicity of FDDs [25].

### Limitations

This study had several limitations. The in vivo experiment did not include any comparison group. Otherwise, the more concentric neointima treated with surface modified FDD would be more convincing. In addition, the small sample size and heterogeneity between blood donors may have affected the in vitro experiment. Nevertheless, further clinical studies are warranted.

## Conclusions

The EMBOPIPE FDD can promote aneurysm occlusion while maintaining branch artery patency. Our pathological findings showed good neointimal coverage of the aneurysm neck, endothelialization along the device, and an intact SMC layer. In addition, the EMBOPIPE was associated with a low degree of thrombus accumulation along the struts of the device. Use of the EMBOPIPE appears to be safe and feasible for the treatment of intracranial aneurysms.

## Data Availability

The primary data of this study are available from the corresponding author on reasonable request.
