# Correction: Evaluation of the EMBOPIPE flow diverter device: in vivo and in vitro experiments

**DOI:** 10.1186/s41016-024-00362-7

**Published:** 2024-04-08

**Authors:** Yongnan Zhu, Fanyan Zeng, Jian Liu, Shiqing Mu, Ying Zhang, Xinjian Yang

**Affiliations:** 1 Department of Beijing Neurosurgical Institute, Fengtai District, Capital Medical University, No. 119, South Fourth Ring West Road, Beijing, 100070 People’s Republic of China; 2 Fengxian District, Heartcare Medical Technology Co., Ltd, Building 38, No. 356 Zhengbo Road, Shanghai, 200000 People’s Republic of China; 3 Neurosurgical Institute & Department of Neurosurgery, Fengtai District, Beijing Tiantan Hospital, Capital Medical University, No. 119, South Fourth Ring West Road, Beijing, 100070 People’s Republic of China

**Correction:** **Chin Neurosurg J 10, 8 (2024)**

**https://doi.org/10.1186/s41016-024-00360-9**

Following publication of the original article [1], the authors reported that the authorship needed to be updated. They want to change the author Xinjian Yang to the main corresponding author and the author Ying Zhang to the co-corresponding author, interchanging the order of the authors Yangxin Jian and Ying Zhang. This error was caused by the negligence of the authors. XinJian Yang mainly contributed to the planning of the project and participated in writing the draft and finalizing the manuscript. The updated authorship has been provided in this Correction.

The original authorship is:

Yongnan Zhu^1^, Fanyan Zeng^2^, Jian Liu^3^, Shiqing Mu^3^, Xinjian Yang^1,3^ and Ying Zhang^1*^

*Correspondence:

Ying Zhang

yingzhang829@163.com

The updated authorship is:

Yongnan Zhu^1^, Fanyan Zeng^2^, Jian Liu^3^, Shiqing Mu^3^, Ying Zhang^1*^and Xinjian Yang^1,3*^

*Correspondence:

Ying Zhang

yingzhang829@163.com

Xinjian Yang

yangxinjian@voiceoftiantan.org

The original article [1] has been corrected.
